# Risk Assessment of Neonatal Exposure to Low Frequency Noise Based on Balance in Mice

**DOI:** 10.3389/fnbeh.2017.00030

**Published:** 2017-02-22

**Authors:** Nobutaka Ohgami, Reina Oshino, Hiromasa Ninomiya, Xiang Li, Masashi Kato, Ichiro Yajima, Masashi Kato

**Affiliations:** ^1^Department of Occupational and Environmental Health, Nagoya University Graduate School of MedicineNagoya, Japan; ^2^Nutritional Health Science Research Center, Chubu UniversityKasugai, Japan; ^3^Department of Electrical and Mechanical Engineering, Nagoya Institute of TechnologyNagoya, Japan

**Keywords:** neonatal exposure, low frequency noise, balance, motor activity, vestibule

## Abstract

General electric devices and ventilation systems are known to generate low frequency noise (LFN) with frequencies of <100 Hz. Previous studies showed that exposure to LFN caused impairments of balance in humans and mice during adulthood. On the other hand, a previous study showed that noise levels in the neonatal intensive care unit (NICU) were greater than those in general home or office environments. Therefore, it is possible that neonates have a potential risk to be exposed to LFN in the NICU. However, the risk of neonatal exposure to LFN remains unclear in humans and mice. In this study, male ICR mice were exposed to LFN at 100 Hz for 4 weeks after birth and then subjected to rotarod and beam crossing tests in order to assess LFN-mediated risk of imbalance during the neonatal period. Exposure to LFN at 70 dB, but not exposure to LFN up to 60 dB, during the neonatal period significantly decreased performance scores for rotarod and beam crossing tests compared to the scores of the control group. The number of calbindin-positive hair cells in the saccule and utricle was decreased in mice exposed to LFN at 70 dB for 4 weeks in the neonatal phase. Cessation of exposure for 10 weeks did not result in recovery of the decreased performance in rotarod and beam crossing tests. Thus, our results suggest that 70 dB is a possible threshold for exposure to LFN for 4 weeks during the neonatal period causing unrecoverable imbalance in mice.

## Introduction

Exposure to audible noise at excessive levels is known to cause noise-induced hearing loss (Dougherty and Welsh, 1966; Wallenius, 2004) but information about the frequency-dependent influence of noise on health is limited. Low frequency noise (LFN) is defined as a sound with frequency below 100 Hz, and infrasound usually has a frequency below 20 Hz (Berger et al., 2015). In our previous study, electric devices used daily were shown to generate LFN with characteristic sounds having a peak at 100 Hz and 70 dB (Tamura et al., 2012). Thus, we are potentially exposed to LFN generated from many devices including public transportation, industrial machines, air circulating devices (e.g., wind fans, ventilation and air-conditioning devices) and household devices (e.g., heat pumps, ventilation fans, washing machines, refrigerators and freezers) in daily and occupational environments.

Exposure to LFN has been shown to affect some physiological functions including functions of the cardiovascular and nervous systems and the endocrine system in humans (Leventhall, 2003; Schust, 2004). Exposure to LFN has also been shown to cause annoyance, sleep disturbance and impairments of wakefulness, perception, evoked potentials and cognition (Karpova et al., 1970; Landstroem et al., 1983). Exposure to infrasound has been shown to cause impairments of blood pressure, leading to hypertension in humans (Danielsson and Landström, 1985). Furthermore, occupational exposure of human adults to LFN at 70 dB has been shown to increase cortisol levels in saliva samples (Waye et al., 2002). A previous study also suggested a risk of maternal stress caused by exposure to noise in humans (Kihal-Talantikite et al., 2013). Thus, previous studies have suggested that LFN can cause health problems in human adults. However, there is no information about the influence of exposure to LFN during the neonatal period on health risks.

The inner ears consist of the organ of Corti, the vestibule and the semicircular canal. The vestibule contains the utricle and saccule, both of which perceive linear acceleration and gravity. In the utricle and saccule, the otolith, a complex of calcium crystal and protein that is located on the hair bundles of hair cells, plays a crucial role in mechanotransduction for balance perception (Lundberg et al., 2006). In an experimental study, behavior analyses including rotarod and beam-crossing tests are usually performed to determine balance in mice. Electrophysiological impairments of hair cells in the utricle have been shown to be involved in imbalance assessed by rotarod analysis in mice (Horwitz et al., 2011). Thus, hair cells in the saccule and utricle play an important role in balance.

In a previous study, occupational exposure to LFN was shown to cause impairments of vestibular functions assessed by a caloric test in human adults (Doroshenko and Stepchuk, 1983). Our previous study showed that exposure to LFN at 70 dB for 4 weeks during adulthood affected scores of rotarod and beam-crossing tests in mice (Tamura et al., 2012). Thus, these previous studies suggest that exposure to LFN during adulthood increases risks of imbalance in humans and mice. However, there is no information about risk assessment of exposure to LFN in a developmental stage. Therefore, we performed experiments in which wild-type mice were exposed to LFN during the neonatal period to assess the risk for imbalance.

## Materials and methods

### Mice

ICR mice and C57BL/6J mice (Japan SLC, Hamamatsu, Japan) were separately bred in a specific pathogen-free (SPF) environment with room temperature at 23 ± 2°C and a 12-h light/dark cycle as previously described (Tamura et al., 2012). All experiments were approved by the Institutional Animal Care and Use Committee in Nagoya University (approval number: 28251) and Chubu University (approval number: 2810030) and followed the Japanese Government Regulations for Animal Experiments.

### Noise exposure

**Figure 1:**
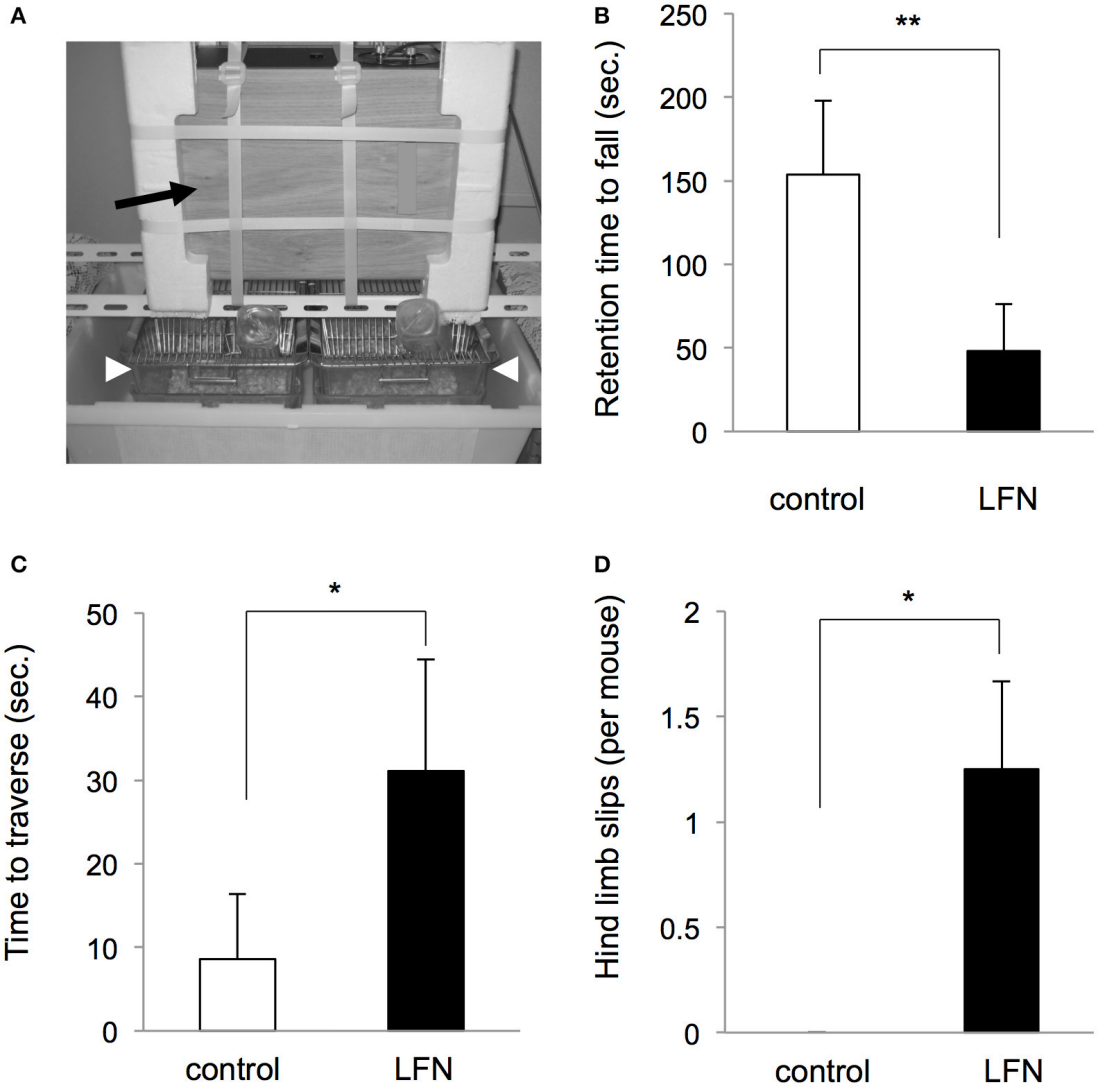
**Rotarod analysis after exposure to LFN for 4 weeks during the neonatal period. (A)** A photograph of the exposure setting of low frequency noise (LFN) for mice. The arrow indicates a speaker and arrowheads indicate breeding cages. **(B–D)** After exposure to LFN at 100 Hz, 70 dB for 4 weeks, **(B)** a rotarod test and **(C,D)** a beam-crossing test were performed at 4 weeks of age. **(B)** Retention time (seconds, mean ± SEM) on the rotarod, **(C)** time to traverse (seconds, mean ± SEM) and **(D)** number of hind limb slips per mouse (mean ± SEM) on the beam were recorded for the control group (*n* = 4) and neonatal exposure group (*n* = 4). Significant difference (^**^p < 0.01; ^*^p < 0.05) from the control group was analyzed by the unpaired *t*-test.

Neonatal mice were continuously exposed to LFN with a peak of 100 Hz at 50, 60 and 70 dB for 4 weeks at a distance of approximately 15 cm from the speaker in a closed soundproof room (Figures 1A and Figures S1). Control groups were maintained under a normal breeding condition in which the background noise level (mean ± SD) at 100 Hz was 42.3 ± 1.1 dB. We output LFN with a setting of 0.1 ms rise-fall, 600 ms interval and 10 ms flat by a sound stimulator (DPS-725, Dia Medical System CO., LTD, Japan). The exposure for 4 weeks after birth was performed in the presence of mother mice and performed without dividing male and female neonates. After the neonatal exposure, we divided male and female mice and further maintained male mice under the normal breeding condition for 10 weeks as the exposure “cessation” group. We regularly monitored the noise output with a noise level meter (Type 6224 with an FFT analyzer, ACO CO., LTD, Japan) as previously described (Tamura et al., 2012).

### Behavior analyses

**Figure 2:**
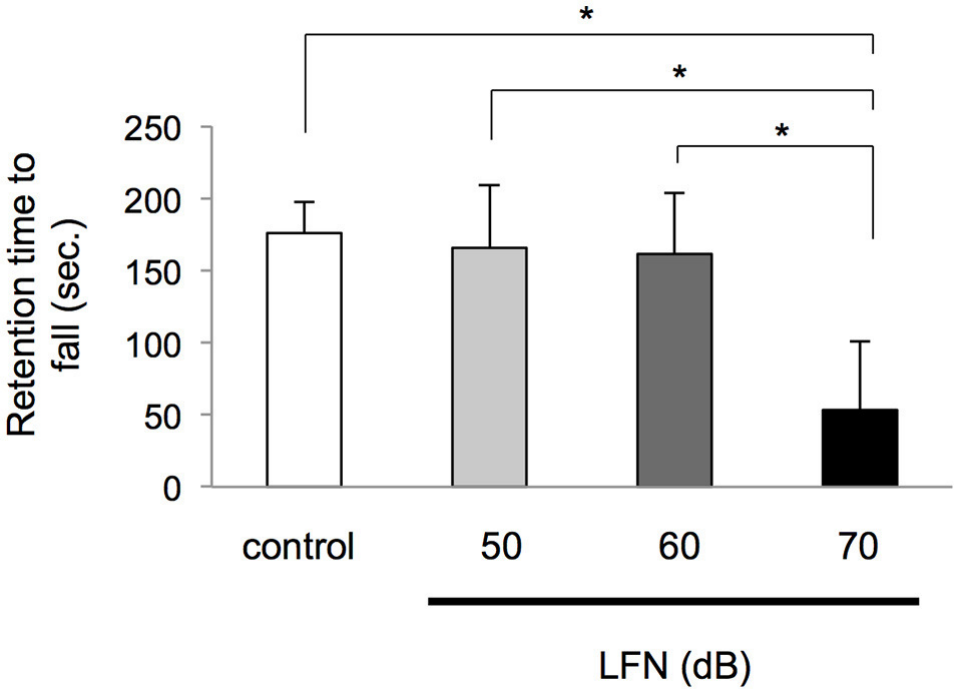
**Exposure to LFN up to 60 dB in the neonatal period did not affect performance on the rotarod**. After exposure to LFN at 100 Hz, 70 dB for 4 weeks, a rotarod test was performed at 4 weeks of age. Retention times (seconds, mean ± SEM) on the rotarod were recorded for the control group and neonatal exposure group at 50, 60, and 70 dB. Four mice per group were tested. Significant differences (^*^p < 0.05) among groups were analyzed by Tukey's *post-hoc* multiple comparison tests.

**Figure 3:**
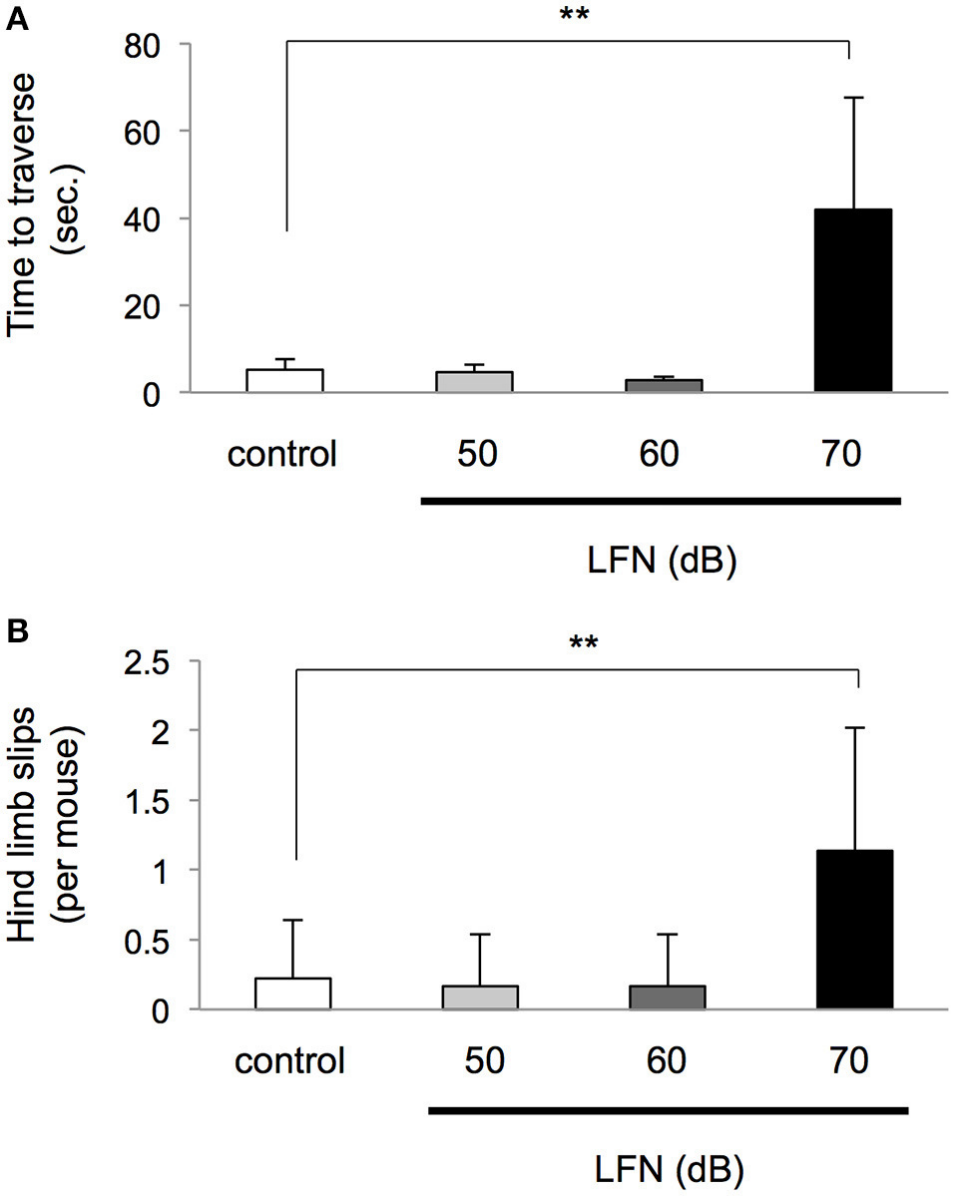
**Exposure to LFN up to 60 dB in the neonatal period did not affect performance on the beam**. After exposure to LFN at 100 Hz, 70 dB for 4 weeks, a beam-crossing test was performed at 4 weeks of age. **(A)** Time to traverse (seconds, mean ± SD) and **(B)** number of hind limb slips per mouse (mean ± *SD*) on the beam were recorded for the control group (*n* = 6) and neonatal exposure group at 50 dB (*n* = 6), 60 dB (*n* = 6) and 70 dB (*n* = 5). Significant differences (^**^p < 0.01) among groups were analyzed by Dunnett's multiple comparison test.

Assessment of balance was performed according to previous studies (Tamura et al., 2012; Tung et al., 2016). We used only male mice for behavior analyses after the neonatal exposure and the exposure cessation to exclude the possibility that the estrous cycle affects behavior analyses in female mice. After the neonatal exposure and the exposure cessation, we examined mice with a rotating rod treadmill (Ugo Basile; Stoelting Co., Chicago, IL). The mice were gently placed into individual lanes of the rotating rod, and the rotating rod test was performed at an acceleration mode (5–40 rpm). We recorded each animal's performance score in seconds when the mouse was unable to continue walking on the rotating rod. Three repeated trials separated by 5-min rests were performed. After a pre-trial, scores of duplicated trials were recorded. We also performed a beam crossing test with a round wooden bar of 2 cm in diameter. We put a beam of 60 cm in length across a container of 40 cm in width, 60 cm in depth and 15 cm in height. We first performed pre-training of mice on the bar at 30 cm in length, followed by three consecutive trials of traversing the bar at 30 cm in length. We set a time limit for 60 s to traverse the beam at 30 cm in length. We recorded the time to traverse and the number of hind limb slips for each mouse. Four mice per group were used for the results shown in Figures 1, 2, and **6** and six mice per group were used for the results shown in Figures 3, **5** except for five mice for the exposure group at 70 dB in Figure 3.

### Immunohistochemistry

Morphological analyses were performed as described previously (Ohgami et al., 2010, 2012a,b). We used three male mice per group for results shown in Figure 1. A total of nine serial sections from three mice per group were used. In brief, perfusion fixation was performed using Bouin's solution and then inner ears from mice were immersed in Bouin's solution for 3 days to 1 week at 4°C. Immunohistochemical analysis with a polyclonal antibody against calbindin D28k (1:200; Santa Cruz, C-20) was performed with paraffin sections. Alexa594 Donkey Anti-Goat IgG (H+L) (Invitrogen, A-11058) was used as a secondary antibody followed by counterstaining with 4′,6-diamidino-2-phenylindole (DAPI). The specimens were observed under a fluorescent microscope (Leica DMI6000B). The software program WinROOF (Mitani Corp., Fukui, Japan) was used for immunohistochemical estimation of positive cells detected by antibodies as previously reported (Ohgami et al., 2016a). A total of 9 serial sections from 3 mice per group were used for the estimation.

### Statistical analysis

**Figure 4:**
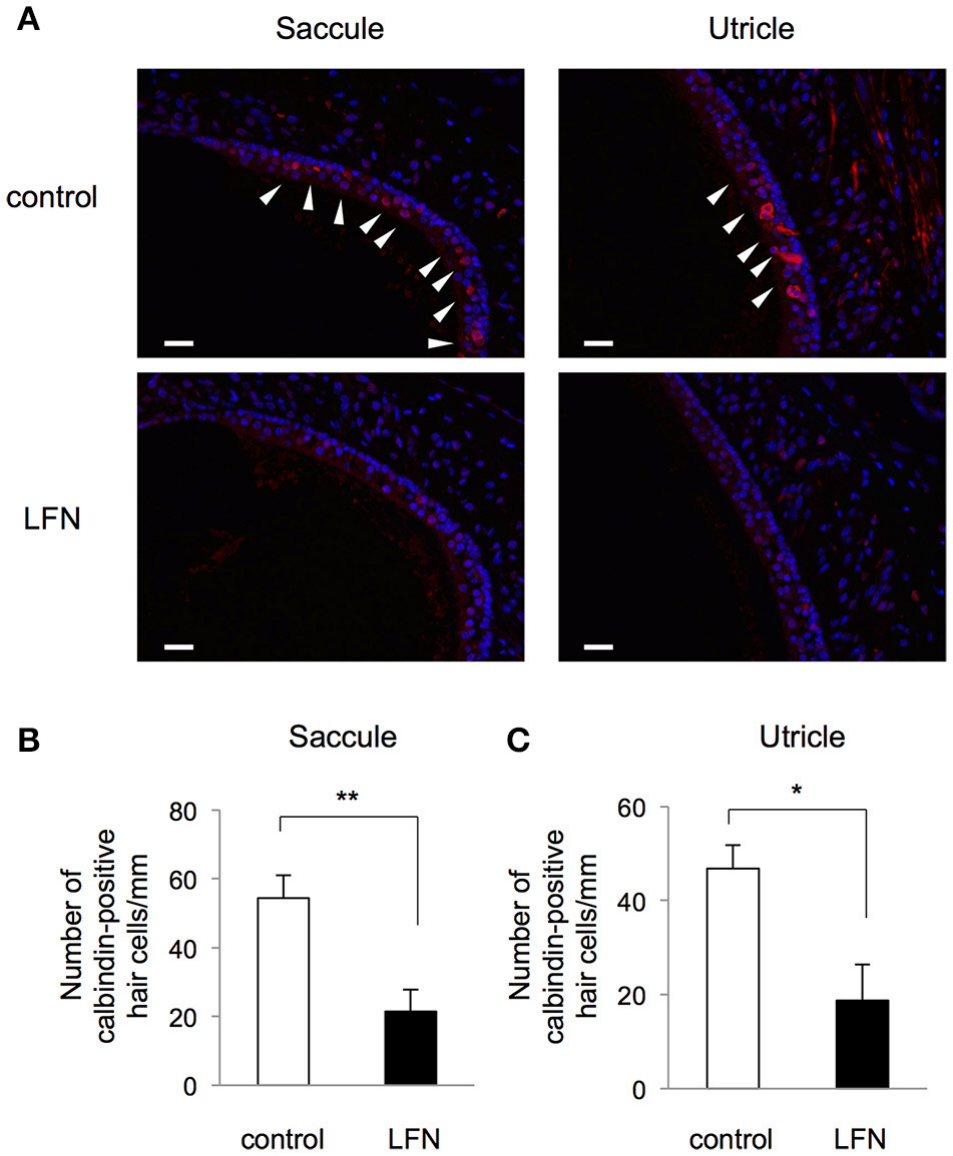
**Exposure to LFN decreased the number of calbindin-positive hair cells in the saccule and utricle. (A)** Immunohistochemistry of hair cells (red) at equivalent positions in the saccule (left panels) and utricle (right panels) from the control group (upper panels) and neonatal LFN-exposure group (lower panels) detected by anti-calbindin D28k antibody. Counterstaining was performed with DAPI (blue). Arrows indicate positive hair cells detected by anti-calbindin D28k antibody. Scale bars: 20 μm. **(B,C)** Number of calbindin-positive hair cells per mm (mean ± SEM) in the saccule **(B)** and utricle **(C)** from a total of nine serial sections from three mice per groups. Results for the LFN-exposure group (closed bars) and control group (open bars) are presented. Significant difference (^**^p < 0.01; ^*^p < 0.05) from the control was analyzed by the unpaired *t-*test.

**Figure 5:**
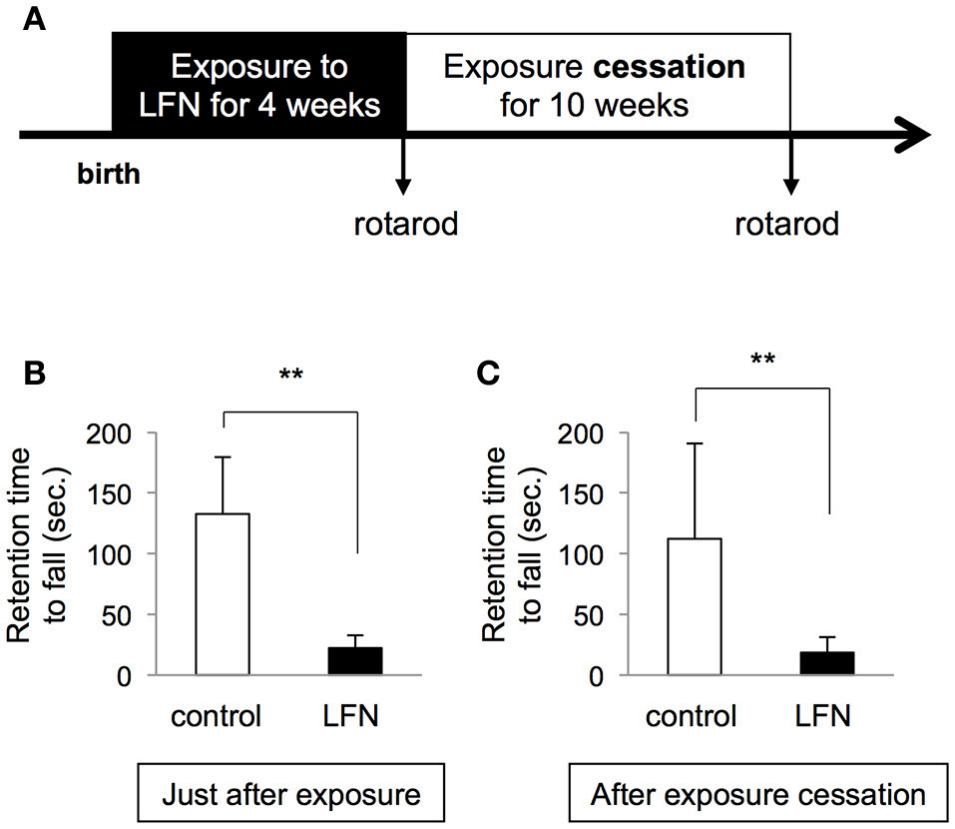
**Exposure to LFN in the neonatal period resulted in irreversible impairment of performance on the rotarod**. **(A)** Scheme of exposure to low frequency noise (LFN) at 70 dB for 4 weeks during the neonatal period and cessation of exposure for 10 weeks. After birth, ICR mice were continuously exposed to LFN at 70 dB for 4 weeks. After the exposure, mice were maintained for 10 weeks under the condition of exposure cessation (in the absence of exposure to LFN). Rotarod tests were performed just after exposure and after exposure cessation. **(B,C)** Retention times (seconds, mean ± *SD*) of each group (*n* = 6) on the rotarod were recorded just after exposure **(B)** and after exposure cessation **(C)**. Results for the LFN-exposure group (closed bars) and control group (open bars) are presented. Significant difference (^**^p < 0.01) from the control was analyzed by the unpaired *t-*test.

**Figure 6:**
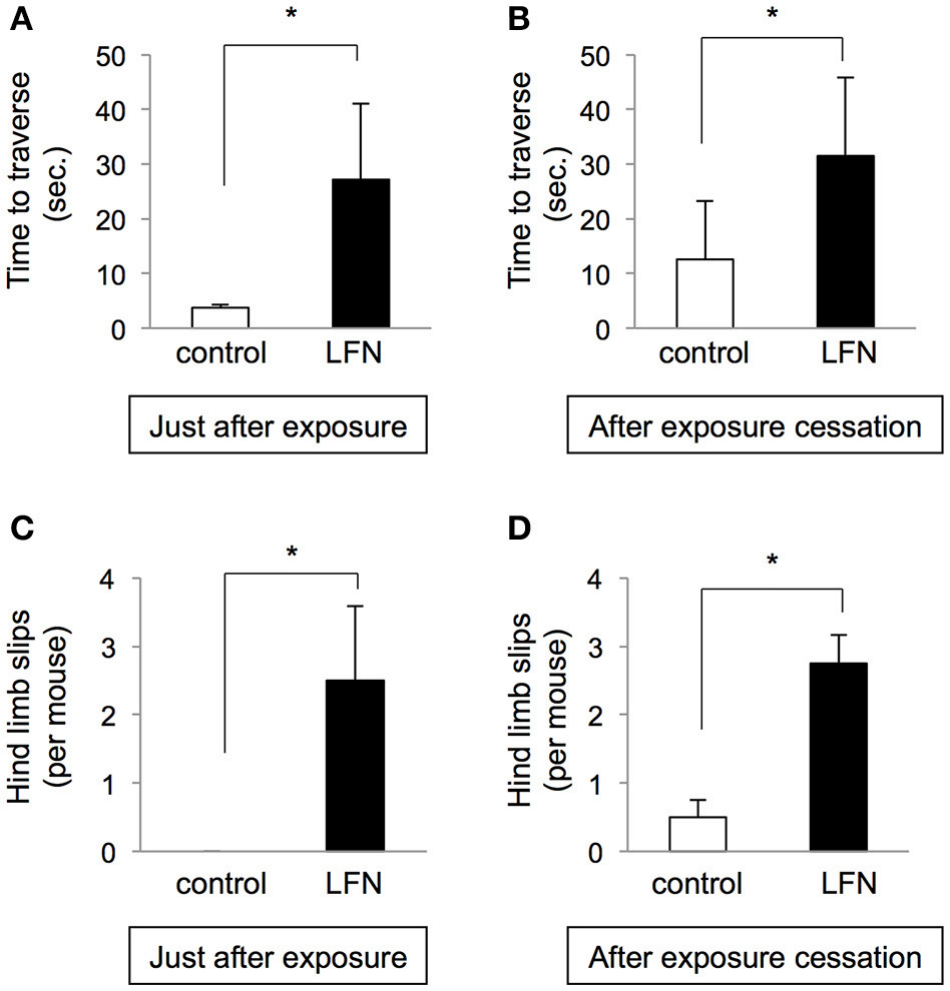
**Exposure to LFN in the neonatal period resulted in irreversible impairment of performance on the beam**. After neonatal exposure to LFN at 70 dB for 4 weeks after birth, mice were maintained for 10 weeks under the condition of exposure cessation. Beam tests were performed just after exposure and after exposure cessation. **(A,B)** Time to traverse (seconds, mean ± SEM) and **(C,D)** number of hind limb slips per mouse (mean ± SEM) of each group (*n* = 4) on the beam were recorded just after exposure **(A,C)** and after exposure cessation **(B,D)**. Results for the LFN-exposure group (closed bars) and control group (open bars) are presented. Significant difference (^*^p < 0.05) from the control was analyzed by the unpaired *t-*test.

Statistical analyses were performed following the methods previously reported (St-Amour et al., 2014; Ohgami et al., 2016b). All statistical analyses were performed by JMP Pro (version 11.0.0; SAS Institute Inc., Cary, NC, USA). The unpaired *t*-test was used to determine a significant difference between two groups (Figures 1, 4–**6**). We used Bartlett's test to assess homogeneity of variances for four groups (Figures 2, 3). In the case of homogeneous variance, one-way ANOVA followed by Tukey's *post-hoc* multiple comparison tests were used to determine significant differences among four groups (Figure 2). When homogeneous variance was denied, Welch's ANOVA followed by Dunnett's multiple comparison tests were used (Figure 3).

## Results

### Exposure to LFN at 70 dB for 4 weeks during the neonatal period affected balance

We performed an experiment with exposure of neonatal mice to LFN with a frequency of 100 Hz for 4 weeks after birth in order to determine whether exposure to LFN during the neonatal period affects balance in mice (Figure 1A). Rotarod analysis showed that exposure of neonatal mice to LFN at 70 dB significantly affected rotarod performance at 1 month of age compared to the performance by the control group (Figure 1B). For mice that had been exposed to LFN in the neonatal period, the time to traverse and the number of hind limb slips in the beam crossing test were significantly increased compared to those of the control group (Figures 1C,D).

### 70 dB is a possible threshold for exposure to LFN for 4 weeks in neonatal mice

We next performed an experiment with exposure of neonatal mice to LFN with a peak of 100 Hz at different noise levels including 50, 60 and 70 dB for 4 weeks (Figure S1) to determine a possible threshold affecting balance. After exposure for 4 weeks after birth, the retention time to fall on the rotarod in the group exposed to LFN at 70 dB, but not the groups exposed to LFN at 50 and 60 dB, significantly decreased compared to that in the control group [*F*_(3, 12)_ = 6.15, *p* = 0.009 by one-way ANOVA; *p* < 0.05 by Tukey's test] (Figure 2). In beam crossing tests, the time to traverse and hind limb slips in neonatal mice exposed to LFN at 70 dB, but not the groups exposed to LFN at 50 dB and 60 dB, were significantly increased compared to those in the control group [*F*_(3, 27)_ = 13.42, *p* < 0.0001 by Welch's ANOVA; *p* < 0.0001 by Dunnett's test] (Figure 3A). Also, hind limb slips in neonatal mice exposed to LFN at 70 dB were significantly increased compared to those in the control group [*F*_(3, 31)_ = 3.77, *p* = 0.020 by Welch's ANOVA; *p* < 0.0001 by Dunnett's test] (Figure 3B).

### Influence of exposure to LFN at 70 dB for 4 weeks during the neonatal period was irreversible

We further performed immunohistochemistry with anti-calbindin D28k, a marker of vestibular hair cells. The results for mice exposed to LFN for 4 weeks during the neonatal period showed decreased numbers of calbindin-positive hair cells in the saccule and utricle compared to those in the control group (Figure 4). We finally examined whether the impairment of balance in neonatal mice exposed to LFN at 70 dB for 4 weeks is reversible (Figure 5A). Rotarod analysis showed decreased retention time to fall in neonatal mice just after exposure to LFN at 70 dB for 4 weeks (Figure 5B). After exposure cessation for 10 weeks, retention time to fall in mice exposed to LFN at 70 dB for 4 weeks during the neonatal period was significantly shorter than that in the control group (Figure 5C). In the beam crossing test, the time to traverse and the number of hind limb steps in mice exposed to LFN at 70 dB for 4 weeks during the neonatal period were significantly increased compared to those in the control group (Figures 6A,C) even after exposure cessation for 10 weeks (Figures 6B,D).

## Discussion

This study is the first study to assess the health risk of exposure to LFN during the neonatal period in ICR mice. The results obtained in this study showed that exposure to LFN at 70 dB for 4 weeks, but not exposure to LFN up to 60 dB, affected balance in neonatal mice. Exposure to LFN at 70 dB for 4 weeks during the neonatal period also resulted in imbalance in C57BL6/J mice in this study (Figure S2). Thus, our results suggest that 70 dB is a possible threshold for exposure to LFN for 4 weeks affecting balance in neonatal mice.

In this study, the imbalance in mice after exposure to LFN at 70 dB for 4 weeks during the neonatal period was irreversible. Since the number of calbindin-positive hair cells in the saccule and utricle was decreased in neonatal mice exposed to LFN in this study, it is possible that morphological impairment of hair cells in the saccule and utricle causes irreversible imbalance. These results partially correspond to the results obtained in our previous study showing that exposure to LFN at 70 dB for 4 weeks led to a decrease in the number of calbindin-positive hair cells in the vestibule (Tamura et al., 2012). Exposure to audible noise has been shown to involve an increase of oxidative stress and loss of hair cells in the organ of Corti (Henderson et al., 2006). Therefore, it is likely that the morphological impairment of hair cells in the saccule and utricle caused by exposure to LFN may involve oxidative stress.

Our results showed that exposure to LFN at 70 dB for 4 weeks, but not exposure to LFN up to 60 dB, during the neonatal period caused impairment of balance in mice. A previous study showed that LFN penetrates the uterus of sheep with attenuation of about 10 dB (Gerhardt et al., 1992). Therefore, it is possible that no influence of exposure to LFN at 70 dB during the fetal period reflects attenuation of exposure levels to fetal mice. Further study is needed to investigate the health risks by exposure to LFN at more than 70 dB during the fetal period. On the other hand, a previous study showed that postnatal care by mother mice affects behaviors of offspring after growing (Francis et al., 2003). Therefore, body weights after neonatal exposure to LFN were measured in this study since the exposure was performed in the presence of mother mice. Exposure to LFN at 70 dB up to 4 weeks did not affect body weights of neonatal mice in this study (Table S1). Thus, it is unlikely that exposure to LFN up to 70 dB affects nurture activities by mother mice, although maternal behaviors of mother mice were not examined in this study. A previous study showed increased levels of corticosterone in serum of offspring cared for by stressed mother mice with increased levels of corticosterone in serum (Moles et al., 2008). It would be worthwhile to investigate the influence on other behaviors and stress-related hormones of offspring and mother mice.

It is known that audible ranges of frequency for humans and mice are about 20–20,000 and 1,000–40,000 Hz, respectively (Heffner and Heffner, 2007), while a previous study showed changes of auditory startle response in mice by stimulation of sound with 375 Hz, which is out of the audible range for mice (Jones et al., 2010). In this study, there was no significant difference of hearing levels between the control group and neonatal LFN-exposed group after exposure to LFN (Figure S3). Thus, our results suggest that exposure to LFN at least at 100 Hz, 70 dB for 4 weeks during the neonatal period affects balance but not hearing in mice.

A previous study showed that noise levels in the neonatal intensive care unit (NICU) were greater than those in general home or office environments, indicating the necessity to reduce noise levels in the NICU (Almadhoob and Ohlsson, 2015). However, there is no information about LFN levels in the NICU and health risks for human neonates, although general electric devices are known to generate LFN. Therefore, it is important to monitor exposure levels of human neonates to LFN to decrease potential risks.

## Author contributions

NO did all the analysis and wrote the paper. RO, HN, and XL performed the animal experiments. MK (5th author) and IY contributed to the noise exposure experiments. MK (7th author) supervised the work and wrote the final version of the manuscript.

## Funding

This study was supported in part by Grants-in-Aid for Scientific Research on Innovative Areas (24108001, Living in space; 16H01639), Scientific Research (A) (15H01743 and 15H02588), (B) (16H02962), and (C) (25460178, 16K08343), Grant-in-Aid for Challenging Exploratory Research (26670525) and center of excellence (COE) Project for Private Universities (Nutritional Health Science Research Center; No. S1201007) from the Ministry of Education, Culture, Sports, Science and Technology (MEXT), Matching Planner Program (MP27115658214) from the Japan Science and Technology Agency (JST), the Japan Health Foundation, the Mitsui & Co., Ltd. Environment Fund, Foundation from Center for Advanced Medical and Clinical Research Nagoya University Hospital, the Mitsubishi Foundation, and the Research Foundation for Health Sciences (The KENKO-KAGAKU Zaidan). The funders had no role in study design, data collection and analysis, decision to publish, or preparation of the manuscript.

### Conflict of interest statement

The authors declare that the research was conducted in the absence of any commercial or financial relationships that could be construed as a potential conflict of interest.
