# Supplementary material for: Risk Assessment of Neonatal Exposure to Low Frequency Noise Based on Balance in Mice
Source: Front Behav Neurosci. 2017 Feb 22;11:30. doi: 10.3389/fnbeh.2017.00030 (PMC5319995; doi:10.3389/fnbeh.2017.00030)
Supplement: Supplementary file 1 [file Table1.docx]

**Supplementary Information**

**Risk assessment of neonatal exposure to low frequency noise**

**based on balance in mice**

Nobutaka Ohgami, Reina Oshino, Hiromasa Ninomiya, Xiang Li, Masashi Kato, Ichiro Yajima and Masashi Kato*

*** Correspondence:** Masashi Kato M.D., Ph.D.: katomasa@med.nagoya-u.ac.jp

**Method**

**Auditory brainstem response (ABR).** ABR measurements (AD Instruments Pty. Ltd.) were performed as described previously (Ohgami et al., 2010; 2016a). The threshold was determined by the appearance of the lowest level of the I wave of ABR by 5-dB stepwise stimulation of tone burst sound from 0 dB to 90 dB. Data are presented as means ± SEM.

**Table S1. Body weights of ICR mice exposed to LFN at 70 dB during the neonatal period.**

|  | Body weight (g) | |
| --- | --- | --- |
|  | No exposure | LFN exposure |
| Exposure for 2 weeks | 12.1 ± 0.4 | 11.9 ± 0.5 |
| Exposure for 4 weeks | 31.7 ± 2.4 | 32.5 ± 2.4 |

We performed an experiment with exposure of neonatal mice to LFN with a frequency of 100 Hz at 70 dB for 4 weeks after birth. Body weights (mean ± SD) of mice were measured at 2 weeks and 4 weeks of age. There was no significant difference between the control group and LFN-exposed group by the unpaired t-test (n=4, each group).
