# Supplementary material for: Risk Assessment of Neonatal Exposure to Low Frequency Noise Based on Balance in Mice
Source: Front Behav Neurosci. 2017 Feb 22;11:30. doi: 10.3389/fnbeh.2017.00030 (PMC5319995; doi:10.3389/fnbeh.2017.00030)
Supplement: Supplementary file 2 [file Image1.PDF]

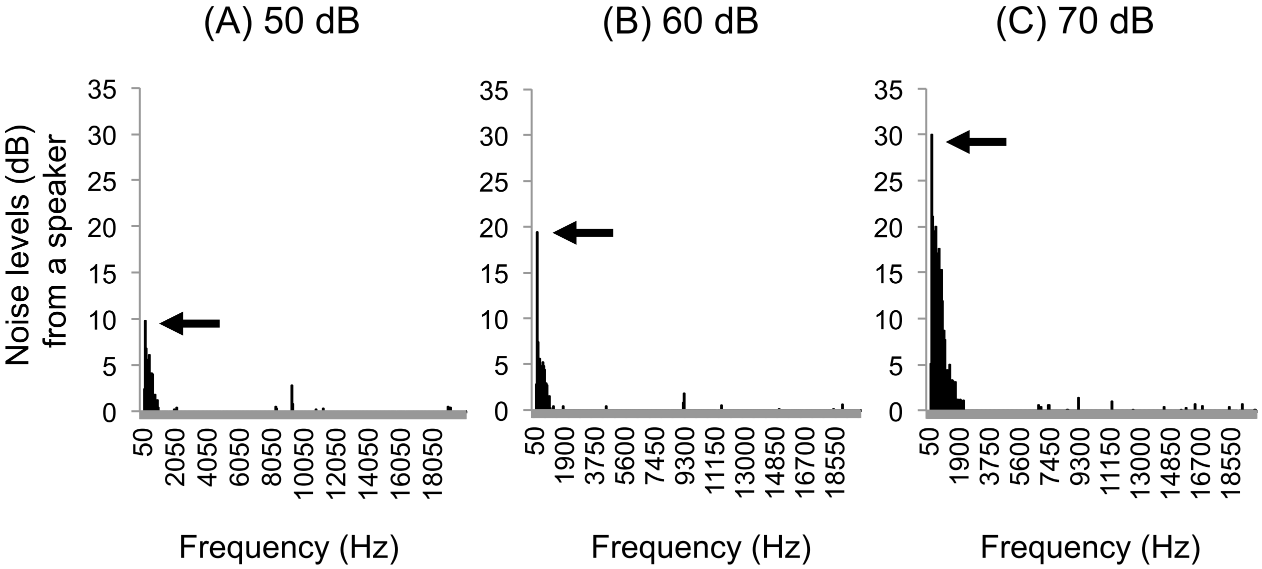

Fig. S1. Sound pattern of LFN used in this study. Sound patterns of low frequency noise (LFN; 100 Hz) at 50 dB (A), at 60 dB (B) and at 70 dB (C) are presented. Arrows indicate peak levels of sound with a frequency of 100 Hz. Noise levels from a speaker at a distance of 15 cm were measured by a noise level meter in a soundproof room. Background levels in the soundproof room were subtracted from noise levels from the speaker.
