# Supplementary material for: Risk Assessment of Neonatal Exposure to Low Frequency Noise Based on Balance in Mice
Source: Front Behav Neurosci. 2017 Feb 22;11:30. doi: 10.3389/fnbeh.2017.00030 (PMC5319995; doi:10.3389/fnbeh.2017.00030)
Supplement: Supplementary file 3 [file Image2.pdf]

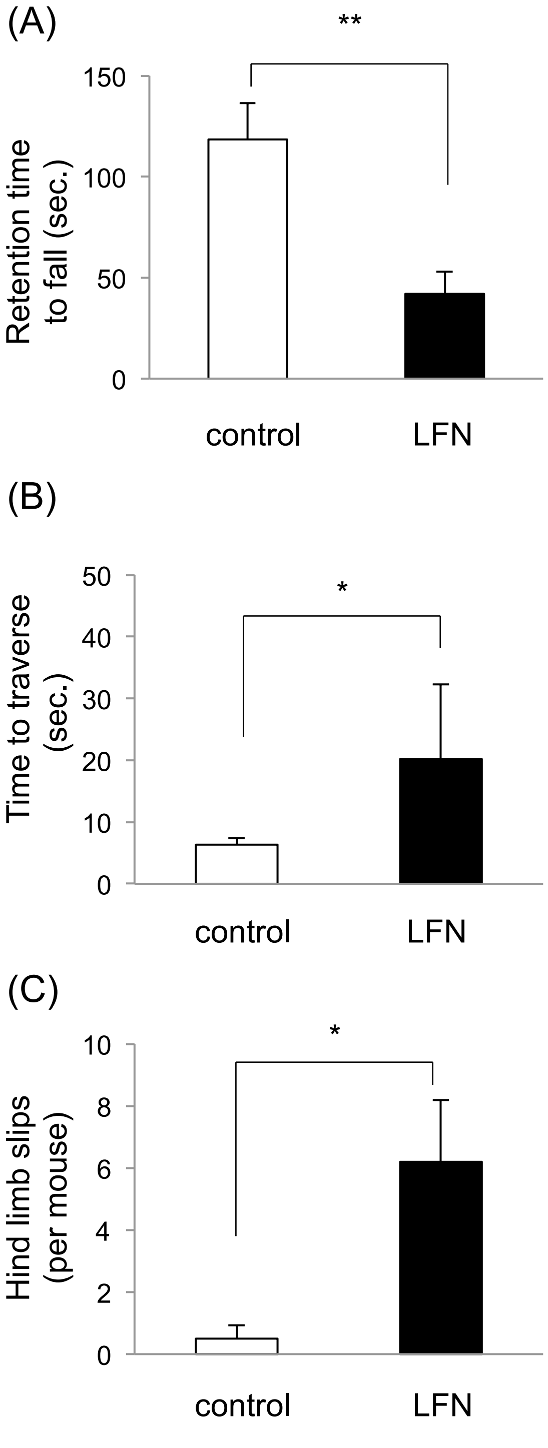

Fig. S2. Rotarod analysis after exposure of C57BL/6J mice to LFN for 4 weeks during the neonatal period. After exposure of C57BL/6J mice to low frequency noise (LFN) at 100 Hz, 70 dB for 4 weeks during the neonatal period, a rotarod test (A) and a beam crossing test (B, C) were performed. (A) Retention time (seconds, mean ± SEM) on the rotarod, (B) time to traverse (seconds, mean ± SEM) and (C) number of hind limb slips per mouse (mean ± SEM) were recorded. Results for the LFN-exposure group (closed bars, n=5) and control group (open bars, n=4) are presented. Significant difference (**, $p < 0.01$; *, $p < 0.05$) from the control was analyzed by the unpaired t-test.
