# Supplementary material for: Risk Assessment of Neonatal Exposure to Low Frequency Noise Based on Balance in Mice
Source: Front Behav Neurosci. 2017 Feb 22;11:30. doi: 10.3389/fnbeh.2017.00030 (PMC5319995; doi:10.3389/fnbeh.2017.00030)
Supplement: Supplementary file 4 [file Image3.pdf]

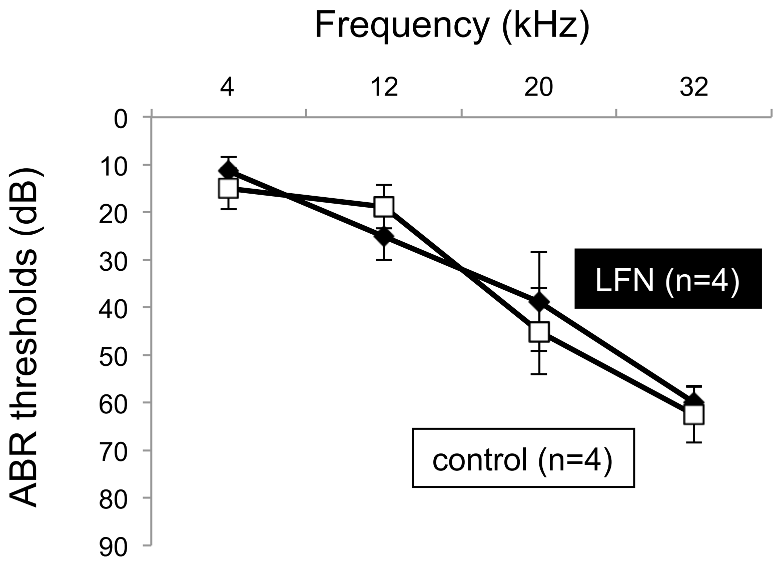

Fig. S3. Influence of neonatal exposure to LFN on hearing in mice. Hearing levels (mean ± SEM) of mice after neonatal exposure to LFN. After exposure of ICR mice to low frequency noise (LFN) at 100 Hz, 70 dB for 4 weeks during the neonatal period, the LFN-exposure group (open squares, n=4) and the control group (closed diamonds, n=4) were used for measuring ABR thresholds.
